# C-954-12. Mental and Social Health Recovery After Massive Burn Injuries

**DOI:** 10.1093/jbcr/irag033.144

**Published:** 2026-04-06

**Authors:** Simrat Kaur, Joseph Grobowski, Caitlin M Orton, Carly Marincasiu, Xinyao deGrauw, Shelley A Wiechman, Gretchen J Carrougher, Julie Hodapp, Haig A Yenikomshian, Lewis E Kazis, Maxwell B Johnson, Karen J Kowalske, Tam N Pham, Barclay T Stewart

**Affiliations:** University of Washington Medicine Regional Burn Center, Harborview Medical Center, Seattle, WA; University of Washington Medicine Regional Burn Center, Harborview Medical Center, Seattle, WA; Department of Surgery, University of Washington Medicine Regional Burn Center, Harborview Medical Center, Seattle, WA; Department of Surgery, University of Washington Medicine Regional Burn Center, Harborview Medical Center, Seattle, WA; University of Washington, Seattle, WA; University of Washington, Harborview Medical Center, Seattle, WA; University of Washington Medicine Regional Burn Center, Harborview Medical Center, Seattle, WA; University of Washington, Seattle, WA; Division of Plastic and Reconstructive Surgery, Keck School of Medicine, University of Southern California, Los Angeles, CA; Boston University School of Public Health, Boston, MA; Division of Plastic and Reconstructive Surgery, Keck School of Medicine, University of Southern California, Los Angeles, CA; Department of Physical Medicine and Rehabilitation, University of Texas Southwest Medical Center, Dallas, TX; University of Washington, Harborview Medical Center, Seattle, WA; Department of Surgery, University of Washington School of Medicine / Harborview Injury Prevention and Research Center, Seattle, WA

## Abstract

**Introduction:**

Survivors of massive burn injury frequently face enduring psychological and social sequelae including anxiety, depression, impaired role function and difficulty with community integration. We aimed to characterize these long-term mental and social health outcomes and compare recovery trajectories across burn size strata (20-49.9%, 50-69.9%, ≥70%). We hypothesized that people living with larger burn sizes would initially report poorer outcomes compared to those with moderate sized burns, but that these deficits would attenuate over time.

**Methods:**

Adults with burns ≥20% TBSA were identified from a multicenter longitudinal cohort and stratified into groups of 20-49.9%, 50-69.9% and ≥ 70% TBSA. Data were collected at discharge (pre-injury recall) and 6, 12, and 24 months post-injury. Mental health was assessed using PROMIS Depression and Anxiety instruments; higher scores indicated worse outcomes. While social health and community integration were evaluated using PROMIS Ability to Participate in Social Roles (APSR) and Community Integration Questionnaire (CIQ); higher scores indicated better outcomes. Demographic and injury characteristics were summarized, and mixed-effects linear regression models were applied to examine longitudinal changes in outcomes.

**Results:**

A total of 816 participants were analyzed. The majority were white (71%), non-Hispanic (78%), and males (76%). Depression and Anxiety scores did not differ significantly at 12 or 24 months compared with 6 months post-injury across all burn size groups and remained slightly above the national population average. APSR scores improved at 12 and 24 months compared to 6 months post-injury in the 20-49.9% TBSA group (Fig. 1; *p*<.05). Participants with ≥70% TBSA reported the lowest APSR across all post-injury timepoints, although changes were not statistically significant. CIQ scores were significantly lower at all timepoints post-injury compared to pre-injury in both the 20-49.9% and 50-69.9% TBSA groups (*p*<.05; Fig. 2).

**Conclusions:**

This analysis details the improving trajectories for mental health, social role participation, and community integration for people living with massive burn injury. Although scores generally improved over time for most burn size groups, people living with burn injury, especially those with the largest injuries, continue to face substantial and enduring challenges.

**Applicability of Research to Practice:**

Findings highlight the chronic psychosocial burden of burn injury, and the need for long-term, targeted support for social and community reintegration. Importantly, people living with massive burn injury generally demonstrate improvement over time across most domains, offering patients and providers reason for optimism regardless of burn size.

**Funding for the study:**

The contents of this abstract were developed under a grant from the National Institute on Disability, Independent Living, and Rehabilitation Research (NIDILRR grant #90DPBU0005). NIDILRR is a Center within the Administration for Community Living (ACL), Department of Health and Human Services (HHS). The contents of this abstract do not necessarily represent the policy of NIDILRR, ACL, HHS, and do not assume endorsement by the Federal Government.

